# β Subunit M2–M3 Loop Conformational Changes Are Uncoupled from α1 β Glycine Receptor Channel Gating: Implications for Human Hereditary Hyperekplexia

**DOI:** 10.1371/journal.pone.0028105

**Published:** 2011-11-22

**Authors:** Qiang Shan, Lu Han, Joseph W. Lynch

**Affiliations:** 1 Queensland Brain Institute, University of Queensland, Brisbane, Queensland, Australia; 2 School of Biomedical Sciences, University of Queensland, Brisbane, Queensland, Australia; Emory University, United States of America

## Abstract

Hereditary hyperekplexia, or startle disease, is a neuromotor disorder caused mainly by mutations that either prevent the surface expression of, or modify the function of, the human heteromeric α1 β glycine receptor (GlyR) chloride channel. There is as yet no explanation as to why hyperekplexia mutations that modify channel function are almost exclusively located in the α1 to the exclusion of β subunit. The majority of these mutations are identified in the M2–M3 loop of the α1 subunit. Here we demonstrate that α1 β GlyR channel function is less sensitive to hyperekplexia-mimicking mutations introduced into the M2–M3 loop of the β than into the α1 subunit. This suggests that the M2–M3 loop of the α subunit dominates the β subunit in gating the α1 β GlyR channel. A further attempt to determine the possible mechanism underlying this phenomenon by using the voltage-clamp fluorometry technique revealed that agonist-induced conformational changes in the β subunit M2–M3 loop were uncoupled from α1 β GlyR channel gating. This is in contrast to the α subunit, where the M2–M3 loop conformational changes were shown to be directly coupled to α1 β GlyR channel gating. Finally, based on analysis of α1 β chimeric receptors, we demonstrate that the structural components responsible for this are distributed throughout the β subunit, implying that the β subunit has evolved without the functional constraint of a normal gating pathway within it. Our study provides a possible explanation of why hereditary hyperekplexia-causing mutations that modify α1 β GlyR channel function are almost exclusively located in the α1 to the exclusion of the β subunit.

## Introduction

Imbalance between the excitatory and inhibitory neurotransmission systems is the cause of many neurological disorders. Human hereditary hyperekplexia (startle disease), which is characterized by exaggerated startle reflexes and hypertonia in response to sudden, unexpected auditory or tactile stimuli, is a neuromotor disorder caused by dysfunction of inhibitory glycinergic neurotransmission in the spinal cord [1]. The majority of genetic mutations identified so far for this disorder have been mapped onto the postsynaptic neurotransmitter receptor, the glycine receptor (GlyR) chloride channel. The synaptic GlyR exists predominantly as the heteromeric α1 β form [2]. However the hereditary hyperekplexia-causing mutations of the GlyR are almost exclusively located in the α1 to the exclusion of β subunit, a fact which has puzzled the field for many years [1], [3], [4].

The GlyR, together with several other postsynaptic neurotransmitter receptors including the nicotinic acetylcholine receptor (nAChR), the type 3 5-hydroxytryptamine receptor (5HT_3_R), and the type A γ-aminobutyric acid receptor (GABAAR), belong to the Cys-loop receptor ligand-gated ion channel superfamily, because they share common structural and functional characteristics [4], [5], [6]. The members of this superfamily exist as pentamers. Each subunit is composed of an N-terminal extracellular domain (ECD) and a transmembrane domain (TMD). The TMD is comprised of four α-helical transmembrane segments (M1–M4) and a large intracellular domain between M3 and M4. Agonists bind to the receptor in a pocket that is formed by the principle (+) and complementary (-) sides of adjacent ECDs. Agonist binding, through a gating pathway, ultimately leads to the opening of a gate in the channel pore, which is formed by the M2 TMDs [7], [8], [9], [10], [11], [12], [13], [14], [15], [16].

**Figure 1:**
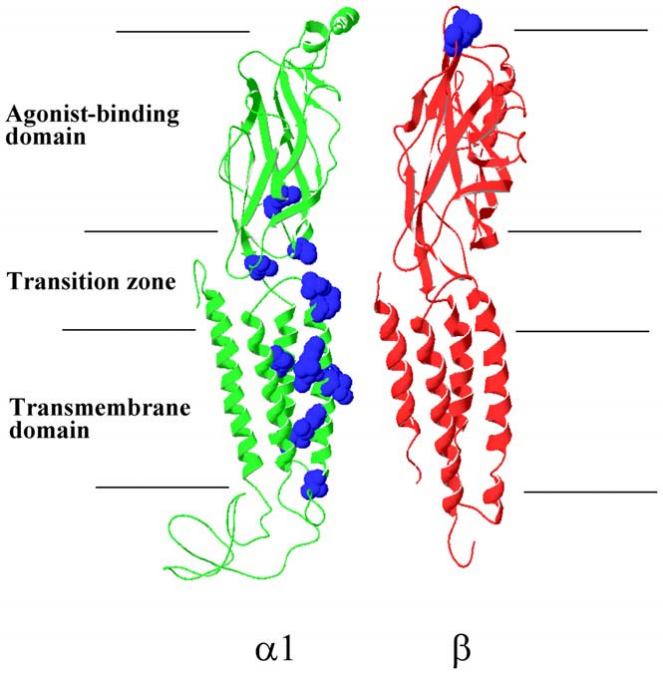
Distribution of hereditary hyperekplexia-causing mutations in the GlyR α1 and β subunits. The hereditary hyperekplexia-causing mutations that disrupt the GlyR channel function rather than block surface expression, are mapped onto the structure models [3] of GlyR α1 (A52S, E103K, R218Q, S231N, I244N, P250T, V260M, T265I, Q266H, S267N, R271L/Q, K276E and Y279C) and β (G229D) subunits.

The putative stoichiometry of the α1 β GlyR is 2α1∶3β [17], although other stoichiometries may also be possible. The α1 β GlyR has been shown to bind the agonist glycine at both the α+/β− and β+/α− subunit interfaces, and agonist binding at either interface is sufficient to activate the channel [17]. Therefore, it seems that the α1 and β subunits play equivalent roles at the agonist-binding level. However, how the α1 and β subunits contribute to the downstream channel gating pathway is barely known. Hyperekplexia-causing GlyR α1 mutations can be classified into two groups: those that disrupt channel function and those that reduce surface expression. Interestingly, most mutations that disrupt GlyR channel function are concentrated within the α1 subunit gating pathway [1], [3] (Fig. 1). Therefore, addressing the question of how the α1 and β subunits contribute to the channel gating pathway is key to solving the puzzle of the predominance of the GlyR α1 mutations in hereditary hyperekplexia.

**Figure 2:**
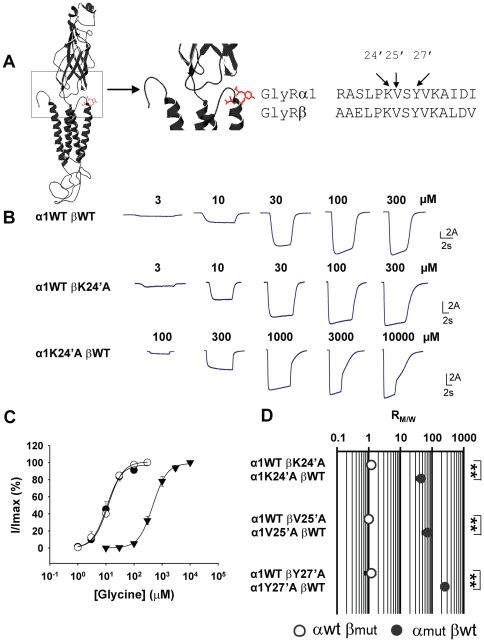
Effects of M2–M3 loop mutations on α1 β GlyR channel function. (A) The positions where mutations were introduced, K24′, V25′ and Y27′, are shown in red in a structure model of the GlyR α1 subunit (left panel). Their positions are also indicated in the amino acid sequences of the M2–M3 loops of the α1 and β subunits (right panel). (B) Example traces of currents induced by increasing glycine concentrations in the indicated receptors. (C) Averaged normalized glycine concentration–response curves for the α1WT βWT GlyR (•),α1WT βK24′A GlyR (○) and the α1K24′A βWT GlyR (▾). (D) The R_M/W_s (the EC_50_ of the mutant α1 β GlyR divided by the EC_50_ of the WT α1 β GlyR) resulting from introducing the K24′A, V25′A or Y27′A mutation into the α1 (•) and β (○) subunits are shown. (** p<0.01 using the Student's t-test).

To address this question, we concentrated on one of the essential structural components of the channel gating pathway, the M2–M3 loop (Fig. 2A). Mutations in this region have been shown to cause drastic effects on channel function in many members of the Cys-loop receptor superfamily [18], [19], [20], [21]. More importantly, this region of the GlyR α1 subunit hosts mutations responsible for most cases of hereditary hyperekplexia, such as R271(19′)Q/L, K276(24′)E, and Y279(27′)C [1], [4], [20]. In addition, a systematic alanine-scanning of this region in the homomeric α1 GlyR further reveals that mutations of a few other residues, notably V277(25′)A, also mimic the phenotype of hereditary hyperekplexia-causing mutations [20]. The primed numbers in brackets after the names of residues refer to the standard M2 domain numbering system that assigns 1′ to the innermost M2 residue. This numbering system will be used henceforth as it enables residues from different subunits to be compared.

In this study, we compared effects of hyperekplexia-mimicking mutations in the M2–M3 loops of α1 and β subunits. We found that α1 β GlyR channel function is less sensitive to mutations introduced into the β than into the α1 subunit. We conclude that the β subunit M2–M3 loop plays a minor role in α1 β GlyR channel gating. A further attempt to identify the possible mechanism underlying this phenomenon indicates that the agonist-induced conformational changes in the β subunit M2–M3 loop are uncoupled from α1 β GlyR channel gating. In addition, we also discovered that the structural components responsible for this are distributed throughout the β subunit, implying that the β subunit has evolved without the functional constraint of a normal gating pathway within it. Our study provides a possible explanation of why hereditary hyperekplexia-causing mutations that modify α1 β GlyR channel function are almost exclusively located in the α1 to the exclusion of the β subunit.

## Results

### α1 β GlyR channel function is less sensitive to hyperekplexia-mimicking mutations introduced into the M2–M3 loop of the β than into the α1 subunit

Previously we reported that α1 β GlyR channel function was not sensitive to cysteine mutations introduced into the M2–M3 loop of the β subunit. We also showed that the channel function of these cysteine-substituted α1 β GlyRs did not change in response to treatment with a cysteine-reactive compound, 2-trimethylammoniumethylmethane thiosulfonate (MTSET) [22]. However, there is a possibility that the cysteine mutations might cause gain-of-function: for example, a disulfide bond could form between the M2–M3 loops of adjacent subunits [23]. In addition, the lack of response to MTSET treatment might be due to the residue not being labeled by MTSET rather than not being sensitive to MTSET modification. We therefore introduced the functionally-inert Ala mutation to verify the results obtained from these experiments.

The following hyperekplexia-mimicking mutations in the M2–M3 loop were introduced, one at a time, to disrupt the channel function: K24′A, V25′A, and Y27′A (Fig. 2A). These mutations, when introduced into the α1 subunit, each cause a dramatic increase in glycine EC_50_ of the homomeric α1 GlyR [20], which in principle could be due to compromised agonist binding, disrupted channel gating, or a mixture of both [24]. As the M2–M3 loop is spatially distant from the agonist binding site based on various structures of Cys-loop receptor members [10], [12], [13], [16] and is temporally downstream from the agonist binding site in the channel gating pathway based on single-channel kinetic analysis [9], the increase in agonist EC_50_ values caused by the K24′A, V25′A, and Y27′A mutations can be attributed predominantly to a disrupted channel gating efficacy. This hypothesis is supported by the results of single-channel kinetic analyses on the M2–M3 loop mutants of both the GlyR and nAChR [25], [26]. Here we use the agonist EC_50_ as an index of channel gating efficacy changes to compare the effects of K24′A, V25′A, and Y27′A mutations, when introduced into the α1 versus β subunits, on channel gating. Similar strategies (using either agonist EC_50_ values or voltages of half activation) have been successfully employed to probe the gating mechanisms of both ligand- and voltage-gated channels [27], [28].

Fig. 2B shows sample currents recorded in response to glycine of increasing concentrations in HEK293 cells expressing α1WT βWT, α1K24′A βWT and α1WT βK24′A GlyRs. The glycine concentration-response curve of the α1K24′A βWT GlyR was dramatically right-shifted relative to that of the α1WT βWT GlyR (Fig. 2C). The α1K24′A βWT GlyR exhibited an EC_50_ of 500±80 µM, which was much higher than the corresponding value, 11±2 µM, recorded in the α1WT βWT GlyR (p<0.01, Table 1). In contrast, the same mutation introduced into the β subunit had no effect on the glycine EC_50_. The concentration-response curve of the α1WT βK24′A GlyR almost overlapped that of the α1WT βWT GlyR (Fig. 2B and C), and the EC_50_ of the α1WT βK24′A GlyR was not significantly different from that of the α1WT βWT GlyR (13±1 µM versus 11±2 µM, p>0.05, Table 1).

**Table 1:** Properties of glycine induced currents of GlyRs recorded in the HEK293 cells.

Constructs	10uM PTX inhibition (%)	100uM PTX inhibition (%)	Glycine EC_50_(µM)	R_M/W_	n_H_	Imax (nA)	n
WT							
αWT βWT	8.5±1.8	33±9	11±2		2.0±0.4	4.3±1.2	4
αWT α-βWT	3.8±2.2	25±2	19±4		1.3±0.3	4.3±0.7	4
αWT β-αWT	8.5±1.9	26±2	33±8		2.4±0.4	8.0±2.3	4
αWT α_B_-βWT	4.8±0.9	33±4	16±3		1.8±0.4	8.6±1.6	5
αWT α_T_-βWT	3.2±0.6	25±6	14±1		2.0±0.1	12.0±1.6	4
19′							
αR19′A βWT	2.8±2.1	20±9	590±70	54 ±11	2.5±0.3	3.9±1.3	4
αR19′A βA19′R	4.2±1.7	22±8	530±120	48 ±14	2.1±0.2	5.5±0.9	5
24′							
αWT βK24′A	6.0±1.2	35±3	13±1	1.2±0.3	1.5±0.3	5.4±1.0	3
αK24′A βWT	2.2±1.1	20±5	500±80	45±12	1.8±0.2	6.3±1.1	4
αWT α-βK24′A	5.6±1.0	35±4	25±4	1.3±0.3	1.5±0.2	8.0±1.8	4
αK24′A α-βWT	4.8±0.7	26±5	83±19	4.3±1.3	1.0±0.1	7.2±0.9	4
αWT β-αK24′A	12.0±3.0	31±3	36±10	1.1±0.4	1.5±0.3	5.9±1.7	4
αK24′A β-αWT	10.0±2.2	23±4	684±157	21±7	2.0±0.3	8.9±1.9	4
25′							
αWT βV25′A	5.7±2.2	36±7	11±1	1.0±0.2	2.0±0.6	7.7±0.8	3
αV25′A βWT	4.7±0.7	38±3	778±144	71±20	1.4±0.2	7.4±2.8	3
αWT α-βV25′A	3.0±1.3	34±4	15±4	0.79±0.26	1.3±0.1	7.6±1.7	4
αV25′A α-βWT	2.9±0.8	21±5	97±27	5.1±1.7	0.92±0.04	8.2±1.4	4
αWT β-αV25′A	8.8±1.6	31±1	33±8	1.0±0.3	2.2±0.3	11.0±1.1	3
αV25′A β-αWT	12.0±2.5	37±6	1097±314	33±12	1.7±0.3	8.9±1.5	4
αWT α_B_-βV25′A	8.9±2.4	43±3	18±2	1.1±0.3	2.2±0.3	9.8±4.9	3
αV25′A α_B_-βWT	4.8±0.6	27±2	186±21	11.6±2.7	1.1±0.0	5.5±0.9	5
αWT α_T_-βV25′A	8.5±1.8	36±3	16±2	1.1±0.2	2.3±0.1	14±3.0	4
αV25′A α_T_-βWT	3.7±1.6	25±4	455±11	33±3	1.4±0.2	6.0±1.7	4
27′							
αWT βY27′A	6.3±0.3	42±2	13±4	1.2±0.4	2.4±0.1	8.0±1.3	3
αY27′A βWT	NA	NA	2780±140	253±57	1.1±0.0	2.0±1.0	4
αWT α-βY27′A	4.9±0.3	39±5	33±9	1.7±0.6	1.3±0.3	6.9±3.1	4
αY27′A α-βWT	NA	NA	660±219	35±13	0.78±0.03	5.7±1.3	4
αWT β-αY27′A	22±7	48±4	88±29	2.7±1.1	2.1±0.6	6.5±1.6	3
αY27′A β-αWT	NA	NA	7286±188	220±51	1.0±0.1	1.3±0.3	4

R_M/W_  =  EC_50_ of mutant GlyR with 24′, 25′ or 27′ Ala substitution divided by EC_50_ of its relevant WT forms without Ala substitution.

NA, Not applicable since no glycine-induced current could be detected in the homomeric αY27′A GlyR.

As noted above, the degree to which channel gating is disrupted is reflected by the increase in agonist EC_50_. Thus, the ratio of mutant to WT glycine EC_50_ (R_M/W_) provides an index of the extent to which channel gating has been disrupted. We will use R_M/W_ in the following text to compare the degrees to which the respective subunits contribute to channel gating. K24′A when introduced into the α1 subunit disrupted the α1 β GlyR channel gating by a factor 45±12, which is the EC_50_ of the α1K24′A βWT GlyR divided by the EC_50_ of the α1WT βWT GlyR (Table 1). On the other hand, K24′A when introduced into the β subunit disrupted the α1 β GlyR channel gating by a factor 1.2±0.3, which is the EC_50_ of the α1WT βK24′A GlyR divided by the EC_50_ of the α1WT βWT GlyR (Table 1).

As summarized in Fig. 2D and Table 1, virtually identical results were obtained for V25′A and Y27′A. We thus conclude that the α1 β GlyR gating efficacy is more affected when the disruption of the gating pathway occurs in the α1 than in the β subunit.

One potential problem in drawing such a conclusion is that there is a possibility that the β subunit was not expressed and that the recorded currents may have arisen from homomeric α1 GlyRs. In such a case, no matter how the α1 and β subunits contribute to the α1 β GlyR channel gating, mutations introduced into the M2–M3 loop of α1 subunit would increase glycine EC_50_, as reported previously [20], and mutations introduced into the β subunit would not affect channel function at all, and give results similar to those we obtained. To eliminate this possibility and to maximize the expression of heteromeric α1 β versus homomeric α1 GlyRs, we transfected cells with α1 and β cDNAs in a ratio of 1∶10. Moreover, we tested the sensitivity of the glycine-induced current to picrotoxin wherever there was a possibility that the recorded current may have arisen from homomeric α1 GlyRs. The heteromeric α1 β GlyR has been shown to be resistant to picrotoxin blockade compared to the homomeric α1 GlyR [29], [30]. The magnitude of picrotoxin blockade can therefore reflect the degree to which the heteromeric α1 β GlyR versus the homomeric α1 GlyR has been expressed. In our experiments, only those cells showing significant picrotoxin resistance (Table 1) were used for further glycine concentration-response investigation. The picrotoxin sensitivity testing was applied only to receptors incorporating α1WT, α1K24′A, and α1V25′A subunits, as no glycine-induced current was detected for the homomeric α1Y27′A GlyR (Table 1).

### β subunit M2–M3 loop conformational changes are uncoupled from α1 β GlyR channel gating

We next sought to determine the mechanism underlying the asymmetrical contributions of the α1 and β subunits to channel gating, i.e. how differently the M2–M3 loops of the α1 and β subunits responded during α1 β GlyR channel gating. To achieve this, we examined conformational changes that the M2–M3 loops of the α1 and β subunits experienced during channel gating by using voltage-clamp fluorometry (VCF). VCF correlates conformational changes occurring at the gate with those occurring in some other domain of interest in real-time [31], [32]. A rhodamine fluorescent dye was used to label the M2–M3 loop, because rhodamine fluorescence exhibits an increase in quantum efficiency as the hydrophobicity of its environment is increased. Thus, rhodamine fluorescence intensity reports local conformational changes that cause a change in its immediate chemical microenvironment. These experiments were carried out in *Xenopus* oocytes as fluorescence detection is not routinely possible in HEK293 cell-expressed GlyRs [32].

Previously we reported that rhodamine methanethiosulfonate (MTSR), when attached to the cysteine-substituted 19′ residue in the homomeric α1 GlyR via a disulfide bond, exhibited an increase in fluorescence intensity upon glycine binding [33]. As the current and fluorescence glycine concentration-response relationships overlapped, we concluded that the fluorophore reported M2–M3 loop conformational changes associated with channel gating.

**Figure 3:**
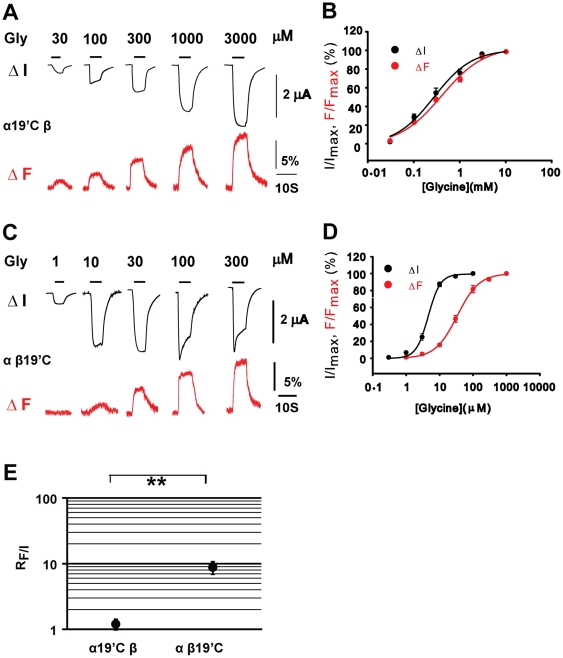
VCF of α1 β GlyRs. Example current and fluorescence traces of the α19′C β and α β19′C GlyRs are shown in (A) and (C), respectively. Averaged normalized glycine concentration-response curves of current and fluorescence of the α19′Cβ and α β19′C GlyRs are shown in (B) and (D), respectively. The R_F/I_s (the EC_50_ of fluorescence divided by the EC_50_ of current) of the α19′C β and α β19′C GlyRs are plotted (E). (** p<0.01 using the Student's t-test).

Cysteine mutations were introduced into either the α1 or β subunits at the 19′ position, and the mutant subunits were co-expressed with the respective WT β or α1 subunits. As shown in Fig. 3A and B, for the α1R19′C β GlyR, where the fluorophore reports conformational changes of the α1 M2–M3 loop, the fluorescence intensity was increased upon glycine application. Moreover, the concentration-response curves of fluorescence and current overlapped and the respective glycine EC_50_ value was not significantly different from each other (329±57 µM and 396±31 µM, respectively, p>0.05, Table 2). This implies that the conformational changes of the α1 M2–M3 loop are coupled to the channel gating in the α1 β GlyR, which is similar to the situation previously demonstrated in the homomeric α1R19′C GlyR [33]. In contrast, in the α1 βA19′C GlyR, where the fluorophore reports conformational changes of the β M2–M3 loop, although the fluorescence intensity was increased upon glycine application as in the α1R19′C β GlyR, the concentration-response curve of the fluorescence was dramatically right-shifted relative to that of the current (Fig. 3C and D). The fluorescence glycine EC_50_ value was 8.8±1.9 times larger than that of current (40.7±8.3 versus 4.61±0.26 µM, p<0.01, Table 2). These data imply that conformational changes of the β M2–M3 loop are uncoupled from channel gating in the α1 β GlyR. The degree of uncoupling is reflected by the ratio of glycine EC_50_ values between fluorescence and current (R_F/I_). These values were 1.2±0.2 and 8.8±1.9 (Fig. 3E and Table 2) for the α1 and β subunits, respectively, which suggests that the gating signal takes the α1 subunit's gating pathway to activate the channel, whereas it bypasses the β subunit's gating pathway.

**Table 2:** Properties of glycine induced currents and fluorescences of MTSR-labeled GlyRs recorded in oocytes.

Constructs	Current			Fluorescence			R_F/I_	n
	EC_50_ (µM)	n_H_	I_max_ (µA)	EC_50_ (µM)	n_H_	F_max_ (%)		
α19′C βWT	329±57	1.20±0.16	3.51±0.24	396±31	0.97±0.04	9.42±0.53	1.2±0.2	8
αWT β19′C	4.61±0.26	2.73±0.38	2.46±0.19	40.7±8.3	1.47±0.12	7.58±0.84	8.8±1.9	10
αWT α-β19′C	4.46±0.43	2.36±0.32	1.53±0.09	6.15±1.08	2.10±0.13	6.58±1.17	1.4±0.3	8
αWT β-α19′C	22.2±1.7	2.26±0.11	3.19±0.08	92.1±5.5	1.15±0.05	3.86±0.37	4.1±0.4	9
αWT α_B_-β19′C	3.82±0.32	2.11±0.11	2.57±0.14	18.0±1.9	1.19±0.12	8.52±1.07	4.7±0.6	7
αWT α_T_-β19′C	2.07±0.23	1.45±0.06	2.19±0.20	14.0±1.0	2.31±0.06	11.5±0.6	6.8±0.9	8

R_F/I_  =  the EC_50_ of fluorescence divided by the EC_50_ of current.

### Structural basis of the lower sensitivity of α1 β GlyR channel function to hyperekplexia-mimicking mutations introduced into the M2–M3 loop of the β than into the α1 subunit

**Figure 4:**
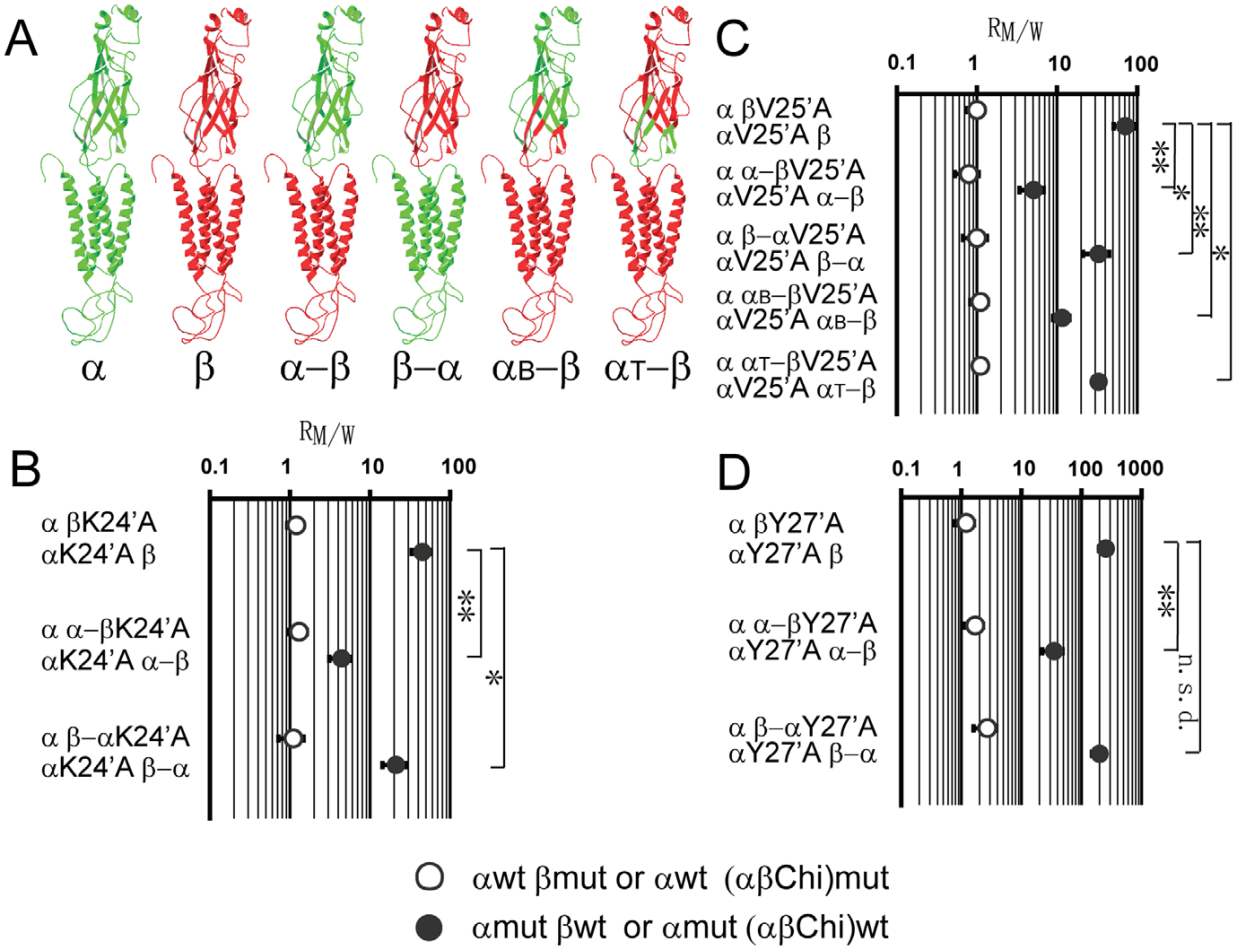
Effects of M2–M3 loop mutations on chimeric α1 β GlyR channel function. (A) The molecular identity of each chimera is schematically illustrated, with green and red denoting α1 and β subunit sequences, respectively. (B–D) The R_M/W_s (the EC_50_ of the mutant chimeric α1 β GlyR divided by the EC_50_ of its relevant WT chimeric α1 β GlyR) resulting from introducing the K24′A, V25′A or Y27′A mutation. The symbol (○) represents constructs containing WT α1 and mutant β or other indicated chimeric subunit, while the symbol (•) represents constructs containing mutant α1 and WT β or other indicated chimeric subunit. (* p<0.05; ** p<0.01; n.s.d. not significantly different; using the Student's t-test).

We investigated whether the minor role of the β subunit in α1 β GlyR channel gating was due to structural differences in the ECD or TMD. To address this question, we constructed two chimeras of α1 and β subunits (Fig. 4A and Fig. S1). Chimera α–β comprises the ECD of the α1 subunit and the TMD (including the M3–M4 domain) of the β subunit. Conversely, chimera β–α comprises the ECD of the β subunit and the TMD of the α1 subunit. We then investigated how these chimeras mimicked the β subunit to contribute to α1 β GlyR channel gating, by co-expressing each chimera with the α1 subunit (10∶1 ratio) and examining the R_M/W_s of the hyperekplexia-mimicking mutations introduced into the α1 and chimeric subunits. It is worth noting that neither chimera, when transfected alone into HEK293 cells, induced any current upon the application of glycine at concentrations up to 100 mM (data not shown).

When the K24′A mutation was introduced, the glycine EC_50_s of the α1K24′A α-βWT and α1WT α-βK24′A GlyRs were 83±19 and 25±4 µM, respectively. The relevant R_M/W_s of the α1 and α-β subunits in the α1 α-β GlyR were 4.3±1.3 and 1.3±0.3 (Fig. 4B and Table 1), respectively. On the other hand, the glycine EC_50_s of the α1K24′A β-αWT and α1WT β-αK24′A GlyRs were 684±157 and 36±10 µM, respectively. The relevant R_M/W_s of the α1 and β-α subunits in the α1 β-α GlyR were 21±7 and 1.1±0.4 (Fig. 4B and Table 1), respectively. These data contrast dramatically with the corresponding values (45±12 and 1.2±0.3) calculated for the α1 β GlyR (Fig. 4B and Table 1). The R_M/W_s of the α1 subunit in both the α1 α-β and α1 β-α GlyRs were significantly less than that of the α1 β GlyR (Fig. 4B, p<0.01 and p<0.05, respectively), suggesting that when the gating pathway is disrupted in the α1 subunit, both the α-β and β-α subunits partially restore channel gating efficacy to that of the WT α1 GlyR. Both the α-β and β-α subunits therefore behave less like the β subunit but more like the α1 subunit. This trend was also found when the other two mutations, V25′A and Y27′A, were investigated in the same way (Fig. 4C, D and Table 1).

It is worth noting that the α-β subunit behaves more like the α1 subunit than does the β-α subunit, based on their abilities to compensate the disrupted gating pathway in the accompanying α1 subunit (Fig. 4B–D). Indeed, the difference in the α1 subunit R_M/W_s between the α1 β-α and α1 β GlyRs was so minor that it was not even significant in the case of the Y27′A mutation (Fig. 4D). Taken together, it seems that the ECD plays a more important role than the TMD in determining the minor role of the β subunit in α1 β GlyR channel gating.

We further dissected the ECD to determine which subdomains contributed to the β subunit's minor role in α1 β GlyR channel gating. The ECDs of the Cys-loop receptors comprise agonist binding sites at subunit interfaces and transition zones, which relay the agonist-binding information to the channel pore. The agonist binding site is formed by the loops A, B, and C from the (+) subunit interface and loops D, E and F from the (-) subunit interface, while the transition zone is formed by loop 2, the conserved Cys-loop and the pre-M1 linker [7], [10], [11], [12], [13], [15], [16]. The agonist binding site and transition zone have been shown to function as relatively independent modules [11]. We therefore investigated which domain might be responsible for the minor role of the β subunit in α1 β GlyR channel gating. To achieve this, two chimeras were constructed. Chimera α_B_-β comprised the α1 subunit agonist binding site and the β subunit transition zone and TMDs, while chimera α_T_-β comprised the α1 subunit transition zone and the β subunit agonist binding site and TMDs (Fig. 4A and Fig. S1). Both chimeras were co-expressed with the α1V25′A subunit, and the ability of each to compensate the disrupted channel gating pathway of the accompanying α1 subunits was examined. The V25′A mutation was investigated here because, among the K24′A, V25′A and Y27′A mutations, the α1V25′A subunit showed the largest difference in R_M/W_ between the α1V25′A β and α1V25′A α-β GlyRs (Fig. 4B–D and Table 1). We therefore expected this mutation would most clearly distinguish the α_B_-β or α_T_-β subunits from the β or α-β subunits when comparing their abilities to compensate the disrupted gating pathway in the accompanying α1 subunits.

As shown in Fig. 4C and Table 1, the glycine EC_50_s of the α1V25′A α_B_-βWT and α1V25′A α_T_-βWT GlyRs were 186±21 and 455±11 µM, respectively. The relevant R_M/W_s of the α1V25′A subunit were 11.6±2.7 and 33±3, respectively, both of which are significantly less than that of the α1V25′A β GlyR (71±20, p<0.01 and p<0.05, respectively). This indicates that both chimeras compensate the disrupted channel gating pathway in the α1V25′A subunit to some degree, but neither of them to the same extent as the α-β subunit (Fig. 4C and Table 1). It is thus evident that both the agonist binding domain and the transition zone of the ECD contribute to the β subunit's minor role in α1 β GlyR channel gating.

### The structural basis of the uncoupling of β subunit M2–M3 loop conformational changes from α1 β GlyR channel gating

As noted above, the reduced sensitivity of α1 β GlyR channel function to hyperekplexia-mimicking mutations introduced into the β subunit was mirrored by the uncoupling of the β M2–M3 loop conformational changes from the channel gating through VCF examination. We next investigated the structural basis for this uncoupling using the same chimera strategy as described in the previous section. We used VCF to monitor conformational changes experienced by the labeled 19′C residues of the α-β, β-α, α_B_-β and α_T_-β subunits when they were co-expressed with the α1 subunit. It is noteworthy that neither glycine-induced current nor fluorescence changes could be detected from any of these chimeric subunits when expressed alone in oocytes (data not shown). Therefore, the fluorescence and current changes we detected when they were co-expressed with the α1 subunit must have arisen from heteromers formed with the α1 subunit.

**Figure 5:**
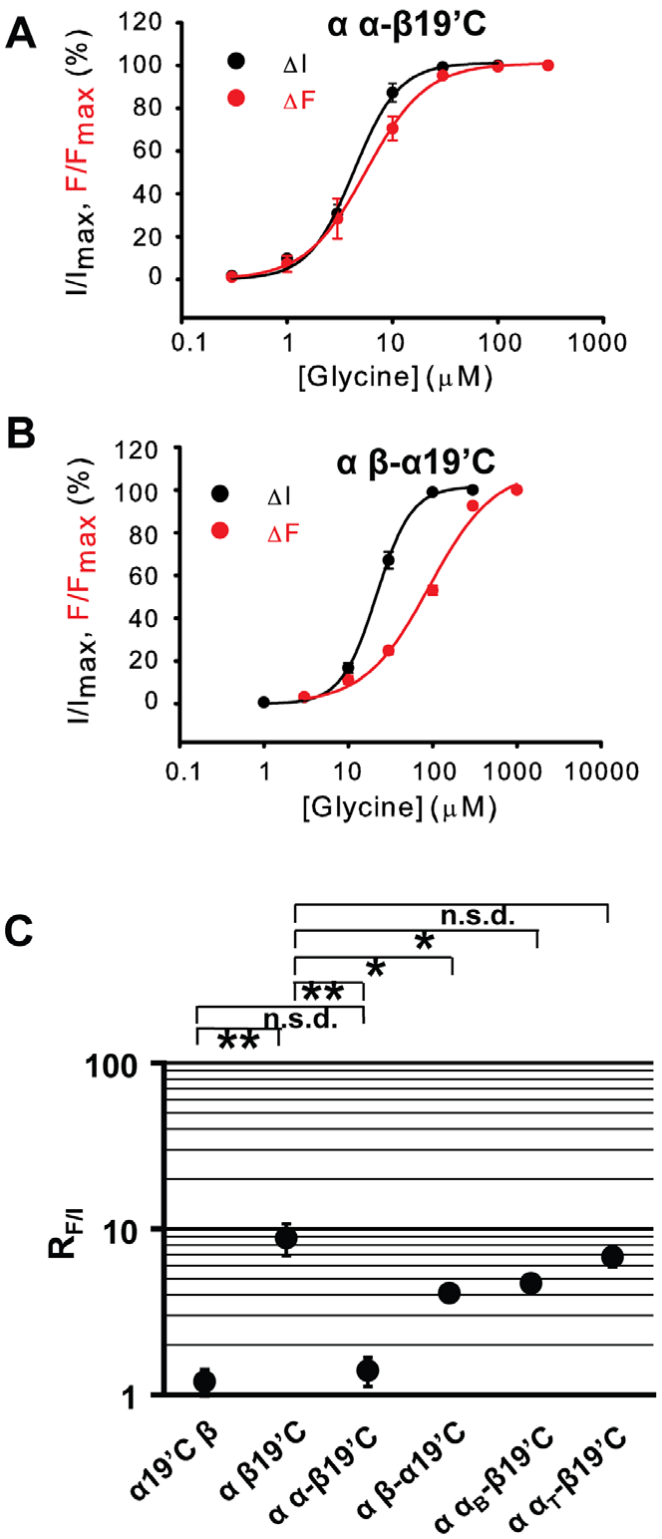
VCF of chimeric α1 β GlyRs. Averaged, normalized glycine concentration-response curves of current and fluorescence of α α-β19′C and α β-α19′C GlyRs are shown in (A) and (B), respectively. (C) The R_F/I_s (the EC_50_ of fluorescence divided by the EC_50_ of current) of the α19′C β, α β19′C, α α-β19′C, α β-α19′C, α α_B_-β19′C and α α_T_-β19′C GlyRs are plotted. (* p<0.05; ** p<0.01; n.s.d. not significantly different, using the Student's t-test).

As shown in Fig. 5A and C, in the α1 α-β19′C GlyR, the fluorescence and current EC_50_ values were 6.15±1.08 and 4.46±0.43 µM, respectively, and the R_F/I_ was 1.4±0.3 (Table 2). On the other hand, in the α1 β-α19′C GlyR, the fluorescence and current EC_50_ values were 92.1±5.5 and 22.2±1.7 µM, respectively, and the R_F/I_ was 4.1±0.4 (Fig. 5B and C and Table 2). The R_F/I_s of both the α-β and β-α subunits were significantly less than that of the β subunit, whose R_F/I_ was 8.8±1.9 (p<0.01 and p<0.05, respectively). This implies that both the ECD and TMD contribute to the uncoupling of the β subunit's M2–M3 conformational changes from the channel gating. The ECD, however, might play a major role since the R_F/I_ of the α-β subunit is not significantly different from that of the α subunit (p>0.05), while the β-α subunit R_F/I_ is closer to that of the β subunit (Fig. 5C). This is consistent with the suggestion that the ECD dominates in determining the minor role of the β subunit in α1 β GlyR channel gating, obtained from the hyperekplexia-mimicking mutation experiments described above.

When further dissecting the ECD, in the α1 α_B_-β19′C GlyR, the fluorescence and current EC_50_ values were 18.0±1.9 and 3.8±0.3 µM, respectively, and the R_F/I_ was 4.7±0.6 (Fig. 5C and Table 2). On the other hand, in the α1 α_T_-β19′C GlyR, the fluorescence and current EC_50_s were 14.0±1.0 and 2.1±0.2 µM, respectively, and the R_F/I_ was 6.8±0.9 (Fig. 5C and Table 2). The R_F/I_s of both chimeras lay between those of the α1 and β subunits (1.2±0.2 and 8.8±1.9, respectively), which implies that both the agonist binding site and transition zone contribute to the uncoupling of the β subunit's M2–M3 conformational changes from the channel gating. It is noteworthy that the transition zone might play a minor role, as the R_F/I_ of the α1 α_T_-β19′C GlyR was not statistically significantly different from that of the α1 β19′C GlyR (p>0.05, Fig. 5C).

## Discussion

### β subunit plays a minor role in α1 β GlyR channel gating

To investigate how the α1 and β subunits each contribute to the channel gating, we assumed that the more a certain subunit contributes to channel gating, the more channel function is compromised when the gating pathway is disrupted in this subunit. By introducing hyperekplexia-mimicking mutations to the M2–M3 loops of the α1 and β subunits, we found that disrupting the channel gating pathway within the α1 subunit had drastic effect on the overall α1 β GlyR function, whereas disrupting the channel gating pathway via the corresponding mutations within the β subunit had little effect. Thus, our results suggest that the α1 subunit dominates channel gating while the β subunit plays only a minor role. Asymmetrical contributions to Cys-loop receptor gating have previously been suggested in the nAChR. For example, cryo-electron microscopic structure analysis shows that the M2 pore-lining domains of the α subunits engage a rotation relative to those of the non-α subunits during channel gating [16], and single channel recording analysis shows a negligible coupling between the pre-M1 linker, the Cys-loop and the M2–M3 loop in the non-α subunits [14]. Our results imply that the α1 subunit of the GlyR behaves like the α subunit of the nAChR, while the β subunit of the GlyR behaves like the non-α subunits of the nAChR.

We employed VCF to investigate the mechanism underlying the asymmetrical contribution of the α1 and β subunits to channel gating. We found that the concentration of agonist required to induce a change in fluorescence of the fluorophore attached to the β subunit is higher than that required to activate the channel. In contrast, the corresponding fluorescence and current concentration-response curves are overlapping when the α1 subunit is labeled. Such an uncoupling of M2–M3 conformational changes from the agonist-induced channel gating has also been demonstrated in the β subunit of the nAChR [34]. A possible explanation for such an uncoupling is that when two or three agonist binding sites are occupied by a low concentration of agonist, the gating pathway is activated along the α1 subunit and the channel is activated. This channel activation might have reached its maximum, since it has been shown that two or three bound agonists are required for full activation of homomeric α1 [35], [36], [37], [38] and heteromeric α1 β GlyRs [39]. In addition, a recent study has shown that in the homomeric α7nAChR-5HT3A receptor, three occupied agonist binding sites at nonconsecutive subunit interfaces are required to exhibit maximal mean channel open time [40]. Therefore, the binding of additional (4th and 5th) agonists when a high concentration of agonist is present, which leads to the gating pathway activation of the β subunit, would not further the channel opening. In other words, a functional β subunit is dispensable, and further disruption of this gating pathway would have no effect on the overall channel gating of the α1 β GlyR.

There is a possibility that fluorescence changes in the α1 β19′C GlyR reflect conformational changes when the channel is desensitized, as both desensitization and fluorescence change appeared only when a high-concentration of glycine was applied (Fig. 3C). However, we consider this is unlikely since fluorescence changes occurred instantly while receptors accumulate in desensitized states with a much slower time course (Fig. 3C).

### Structural basis of the minor role of the β subunit in α1 β GlyR channel gating

By testing chimeras constructed from the α1 and β subunits, we found that both the ECD and TMD were responsible for the β subunit's minor role in α1 β GlyR channel gating (Fig. 4 and 5), although it seems that the ECD plays a dominant role. We originally suspected that the 19′ residue might be the cause since this residue is an Ala in the β subunit (Fig. 2A) and, in the α1 subunit, the R19′A mutation has been shown to drastically compromise α1 GlyR channel function and mimic the phenotype of hyperekplexia-causing mutations [20]. However, when we introduced the A19′R mutation into the β subunit, the contribution of the β subunit to the α1 β GlyR channel gating was not changed (Table 1). Indeed, the natural existence of the 19′A residue in the β subunit might be explained by the fact that, since the β subunit M2–M3 loop is not involved in channel gating and hence not sensitive to mutations, whether a gating-favorable 19′R or gating-disfavorable 19′A exists in the β subunit makes no difference to α1 β GlyR channel gating. Instead, the ECD, which is upstream from the M2–M3 loop in the gating pathway, plays a major role in limiting the contribution of the β subunit to channel gating.

When further dissecting the role of the ECD, our chimera studies indicate that motifs in both the agonist binding site and transition zone determine the contribution of the β subunit to overall receptor gating (Fig. 4 and 5). Thus, no single domain is responsible for the minor role of the β subunit in α1 β GlyR channel gating. One possible explanation is that residues contributing to its minor role are distributed throughout the β subunit, including the agonist binding site, the transition zone and the transmembrane channel pore domains (Fig. 4 and 5). As a result, no single domain from the α1 subunit is able to completely rescue the gating contribution of the β subunit to the level of the α1 subunit.

From an evolutionary perspective, we suggest that the reason why residues that disrupt the β subunit gating are distributed evenly throughout its coding region is that the gating pathway within the β subunit was not optimized to a “normal level” as in the α1 subunit, when the β subunit joined the α1 subunit to form the heteromeric α1 β GlyR. It has been speculated that ancestral Cys-loop receptors were homomers [41], [42] and that the ancestral GlyR might exist in the homomeric α form. Alternatively, even if the gating pathway of the β subunit was equivalent to that of the α1 subunit when the heteromeric α1 β GlyR came into being, random mutations that compromise its channel gating pathway could have accumulated throughout the β subunit during evolution. This is because a normal gating pathway within the β subunit is dispensable for α1 β GlyR channel function and it would not serve as a constraint on the β subunit during evolution. Thus, no single domain from the α1 subunit could rescue β subunit gating efficacy.

The GlyR β subunit is reminiscent of the non-α muscle nAChR subunits, which have much higher ratios of the number of nonsynonymous substitutions to that of synonymous substitutions than the α muscle nAChR subunit, implying less functional constraint on the non-α than α nAChR subunits during evolution [43]. Another example is the AChBP, whose acetylcholine binding but not gating pathway is the function subjected to evolutionary pressure. When the AChBP is connected to the 5HT3R TMD, a functional channel can be formed only if the disabled gating pathway components in the AChBP are replaced by the corresponding ones from the 5HT3R [11].

### Implications for the distribution of hereditary hyperekplexia-causing mutations in the α1 β GlyR

GlyR hereditary hyperekplexia-causing mutations have been mapped almost exclusively onto the gene of the α1 to the exclusion of the β subunit (Fig. 1). This is possibly because the gene of the α1 subunit is a hot spot, more amenable to genetic mutations than that of the β subunit, but it seems more likely because mutations occurring in the α1 subunit more drastically affect α1 β GlyR channel function than those occurring in the β subunit. This assumption is supported by our experiments showing that hyperekplexia-mimicking mutations introduced into the β subunit have much less effect on α1 β GlyR channel function than those introduced into the α1 subunit.

More interestingly, most mutations identified on the α1 subunit that affect channel function (rather than surface expression), cluster either in domains associated with the channel gating pathway (i.e., the M2–M3 loop, loop 2 and the pre-M1 linker) or along the pore-lining M2 domain, but rarely occur in the agonist binding sites (Fig. 1) [1], [3], [4]. This can be explained by the fact that the α1 β GlyR has five potential agonist binding sites and only two or three functional sites are required for efficient gating [17], [39]. Thus, introducing mutations into the agonist binding sites of either the α1 or β subunit will have no effect on overall α1 β GlyR function [17]. In other words, the α1 and β subunits can compensate each other at the agonist binding level, but this compensation between the α1 and β subunits does not pass on to the downstream channel gating pathway. In summary, our experiments provide a possible explanation of why hereditary hyperekplexia-causing mutations concentrate in the channel gating pathway of the α1, to the exclusion of the β subunit, in the α1 β GlyR.

## Materials and Methods

### Mutagenesis and chimera construction of the GlyR cDNAs

The human GlyR α1 and β subunit cDNAs were subcloned into the pcDNA3.1zeo+ (Invitrogen) or pGEMHE [44] plasmid vectors for expression in HEK293 cells or *Xenopus* oocytes, respectively. Site-directed mutagenesis and chimera construction were performed using the QuickChange (Stratagene, La Jolla, CA, USA) mutagenesis and multiple-template-based sequential PCR protocols, respectively.

The multiple-template-based sequential PCR protocol for chimera construction was developed in our laboratory and has recently been described in detail elsewhere [45]. This procedure does not require the existence of restriction sites, or the purification of intermediate PCR products, and needs only two or three simple PCRs followed by general subcloning steps. Most importantly, the chimera joining sites are seamless and the success rate for construction is nearly 100%. The joining sites used in our experiment were chosen based on the following principles: (1) A site, based on the crystal structure of the AChBP [7], is to be near the boundary between the two flanking loops to minimize disturbance on the loop structures. (2) The pair of residues of a joining site is to be conserved between the GlyR α and β subunits, wherever possible. The joining sites used in our experiment are between the following pairs of residues: α L135–T136 and β I157–T158 for the N-terminus of the Cys-loop, α Q155–L156 and β Q178–L179 for the C-terminus of the Cys-loop, α T208-C209 and β T232-C233 for the N-terminus of the pre-M1 linker, and α R218-Q219 and β R242-Q243 for the C-terminus of the pre-M1 linker (Fig. S1). The α R218-Q219 and β R242-Q243 are also the joining sites for chimeras constructed between the ECD and TMD. The loop 2 transposition was achieved by incorporating either the αA52Q or βQ73A mutations, as the loop 2 sequences between the α1 and β subunits are otherwise conserved.

For the VCF experiments, both GlyR α1 and β subunit cDNAs in the pGEMHE vector were mutated to substitute non-essential background cysteines with alanines, including α1C41A and βC115AC291A [46], [47].

For the β-α chimeras used for determining the effect of hyperekplexia-mimicking mutations on α1 β GlyR channel function, the Thr at the M2 6′ position was replaced by a Cys. Thr to Cys mutation at this site in the homomeric α1 GlyR does not affect glycine activation, but does confer picrotoxin resistance on the channel [30]. Through such a modification, picrotoxin resistance was used to distinguish the heteromeric α1 β-α GlyR from the homomeric α1 GlyR when the α1 and β-α subunits were co-expressed.

### HEK293 cell culture, expression and electrophysiological recording

The effects of the hyperekplexia-mimicking mutations, K24′A, V25′A and Y27′A, were examined on GlyRs expressed in HEK293 cells (ATCC). Details of the HEK293 cell culture, GlyR expression and electrophysiological recording of the HEK293 cells are described elsewhere [30]. Briefly, HEK293 cells were maintained in DMEM supplemented with 10% fetal bovine serum. Cells were transfected using a calcium phosphate precipitation protocol. When co-transfecting the α1 together with the β or any other chimera subunits, their respective cDNAs were combined in a ratio of 1∶10. In addition, the pEGFP-N1 (Clontech) was co-transfected to facilitate identifying the transfected cells. Glycine-induced currents were measured using the whole cell patch-clamp configuration. Cells were treated with external Ringer's solution and internal CsCl solution [30]. Cells were voltage-clamped at −40 mV. When the heteromeric GlyRs were expressed, the picrotoxin sensitivity was tested to confirm that the majority of receptors are heteromers [29], [30]. A 10 µM or 100 µM concentration of picrotoxin was applied to the heteromeric GlyRs in the presence of glycine at the EC_50_ concentration of their corresponding α1 homomers. Only the cells with significant picrotoxin resistance compared with their α1 homomers, e.g. where 100 µM picrotoxin inhibited the current by less than 50%, were used for further glycine-sensitivity examination.

### Oocyte preparation, expression and VCF recording

VCF experiments were performed on GlyRs expressed in *Xenopus laevis* oocytes. Female *Xenopus laevis* frogs were purchased from Xenopus Express, France. Details of oocyte preparation, GlyR expression and VCF recording are described elsewhere [33]. Briefly, the mMessage mMachine kit (Ambion, Austin, TX) was used to generate capped mRNA. The mRNA was injected into oocytes of the female *Xenopus laevis* frog with 10 ng (1 ng α1 and 9 ng β or any other chimeric subunits) per oocyte. After injection, the oocytes were incubated in ND96 solution [33] for 3–4 days at 18 °C before recording.

The sulfhydryl-reactive reagent, sulforhodamine methanethiosulfonate (MTSR, Toronto Research Chemicals, North York, Ontario, Canada), was used to label 19′C residues. On the day of recording, the oocytes were labeled with 10 µM MTSR for 25 s, either in the absence or presence of glycine. The oocytes were then transferred to the recording chamber and perfused with ND96 solution. The current was recorded by the two-electrode voltage clamp configuration and the recording electrode was filled with 3 M KCl. Cells were voltage-clamped at −40 mV. The fluorescence was recorded using the PhotoMax 200 photodiode detection system (Dagan Corp., Minneapolis, MN).

### Data analysis

Results are expressed as mean±standard error of the mean of three or more independent experiments. The empirical Hill equation, fitted by a non-linear least squares algorithm (SigmaPlot 9.0, Systat Software, Point Richmond, CA), was used to calculate the EC_50_ and Hill coefficient (n_H_) values for glycine-induced current and fluorescence change. Statistical significance was determined using the Student's t-test.

## Supporting Information

Figure S1 **Amino acid sequence alignment between the human GlyR α1 and β subunits.** The joining sites for chimera construction are highlighted in blue. The K24′, V25′ and Y27′ residues, where hyperekplexia-mimicking mutations were introduced, are highlighted in red. The α1R19′ and βA19′ residues, where the Cys mutation was introduced for VCF experiment, are highlighted in green.
(DOC)
