# Supplementary material for: β Subunit M2–M3 Loop Conformational Changes Are Uncoupled from α1 β Glycine Receptor Channel Gating: Implications for Human Hereditary Hyperekplexia
Source: PLoS One. 2011 Nov 22;6(11):e28105. doi: 10.1371/journal.pone.0028105 (PMC3222680; doi:10.1371/journal.pone.0028105)
Supplement: Figure S1 — Amino acid sequence alignment between the human GlyR α1 and β subunits. The joining sites for chimera construction are highlighted in blue. The K24′, V25′ and Y27′ residues, where hyperekplexia-mimicking mutations were introduced, are highlighted in red. The α1R19′ and βA19′ residues, where the Cys mutation was introduced for VCF experiment, are highlighted in green. (DOC) [file pone.0028105.s001.doc]

GlyRa1 -ARSATKP-------MSPSDFLDKLMGRTSG-------------YDARIRPNFKGPPVNV 39

GlyRb KEKSSKKGKGKKKQYLCPSQQSAEDLARVPANSTSNILNRLLVSYDPRIRPNFKGIPVDV 60

:*:.* :.**: : :.*... **.******** **:*

Loop2

GlyRa1 SCNIFINSFGSIAETTMDYRVNIFLRQQWNDPRLAYN-EYP-DDSLDLDPSMLDSIWKPD 97

GlyRb VVNIFINSFGSIQETTMDYRVNIFLRQKWNDPRLKLPSDFRGSDALTVDPTMYKCLWKPD 120

********** **************:****** :: .*:* :**:* ..:****

Cys-loop

GlyRa1 LFFANEKGAHFHEITTDNKLLRISRNGNVLYSIRITLTLACPMDLKNFPMDVQTCIMQLE 157

GlyRb LFFANEKSANFHDVTQENILLFIFRDGDVLVSMRLSITLSCPLDLTLFPMDTQRCKMQLE 180

*******.*:**::* :* ** * *:*:** *:*:::**:**:**. ****.* * ****

Pre-M1 linker

GlyRa1 SFGYTMNDLIFEWQEQGAVQVADGLTLPQFILK-EEKDLRYCTKHYN-TGKFTCIEARFH 215

GlyRb SFGYTTDDLRFIWQSGDPVQLEK-IALPQFDIKKEDIEYGNCTKYYKGTGYYTCVEVIFT 239

***** :** * **. ..**: . ::**** :* *: : ***:*: ** :**:*. *

GlyRa1 LERQMGYYLIQMYIPSLLIVILSWISFWINMDAAPARVGLGITTVLTMTTQSSGSRASLP 275

GlyRb LRRQVGFYMMGVYAPTLLIVVLSWLSFWINPDASAARVPLGIFSVLSLASECTTLAAELP 299

*.**:*:*:: :* *:****:***:***** **:.*** *** :**:::::.: *.**

GlyRa1 KVSYVKAIDIWMAVCLLFVFSALLEYAAVNFVSRQHK--ELLRFRRKRRHHKEDEAGEGR 333

GlyRb KVSYVKALDVWLIACLLFGFASLVEYAVVQVMLNNPKRVEAEKARIAKAEQADGKGGNVA 359

*******:*:*: .**** *::*:***.*:.: .: * * : * : .: :.:.*:

GlyRa1 FNFSAYGMGP----ACLQAKDG----------------ISVKGAN--------------- 358

GlyRb KKNTVNGTGTPVHISTLQVGETRCKKVCTSKSDLRSNDFSIVGSLPRDFELSNYDCYGKP 419

: :. * *. : **. : :*: *:

GlyRa1 ---NSNTTNPPPAPSKSPEEMRKLFIQRAKKIDKISRIGFPMAFLIFNMFYWIIYKIVRR 415

GlyRb IEVNNGLGKSQAKNNKKPPPAKPVIPTAAKRIDLYARALFPFCFLFFNVIYWSIYL---- 475

*.. :. . .*.* : :: **:** :* **:.**:**::** **

GlyRa1 EDVHNQ 421

GlyRb ------
